# Development of a High-Hydrostatic-Pressure-Treated Recombinant Vaccine Targeting the Major Capsid Protein of Red Sea Bream Iridovirus

**DOI:** 10.3390/ijms27020675

**Published:** 2026-01-09

**Authors:** Yuta Sawasaki, Shogo Harakawa, Shin-Ichi Kitamura, Naomi Terawaki, Zhangliang Zhu, Kohdai Yamada, Hinako Fujisaki, Suzuno Hirano, Mana Hamada, Takuya Miyakawa, Tomomasa Matsuyama, Yuta Matsuura, Tatsuhiko Ozawa, Tomokazu Itano, Tatsuya Sawasaki, Akira Nozawa

**Affiliations:** 1 Proteo-Science Center, PIAS, Ehime University, 3 Bunkyo-cho, Matsuyama 790-8577, Ehime, Japan; k815001h@mails.cc.ehime-u.ac.jp (Y.S.); terawaki.naomi.zu@ehime-u.ac.jp (N.T.); yamada.kodai.pi@ehime-u.ac.jp (K.Y.); n803024y@mails.cc.ehime-u.ac.jp (H.F.); m803033c@mails.cc.ehime-u.ac.jp (S.H.); m803031k@mails.cc.ehime-u.ac.jp (M.H.); 2 Ehime Fisheries Research Center, Ehime Research Institute of Agriculture, Forestry and Fisheries, 309-4, Sakashizu, Uwajima 798-0087, Ehime, Japan; harakawa-syougo@pref.ehime.lg.jp (S.H.); itano-tomokazu@pref.ehime.lg.jp (T.I.); 3 Graduate School of Bioresources, Mie University, 1577 Kurimamachiya-cho, Tsu 514-8507, Mie, Japan; kitamura@bio.mie-u.ac.jp; 4 Graduate School of Biostudies, Kyoto University, Kitashirakawa-Oiwake-cho, Sakyo-ku, Kyoto 606-8502, Kyoto, Japan; zhu.zhangliang.8i@kyoto-u.ac.jp (Z.Z.); miyakawa.takuya.7j@kyoto-u.ac.jp (T.M.); 5 Japan Fisheries Research and Education Agency, Pathology Division, Aquaculture Research Department, Fisheries Technology Institute, Minami-Ise 516-0193, Mie, Japan; matsuyama_tomomasa55@fra.go.jp (T.M.); matsuura_yuta53@fra.go.jp (Y.M.); 6 Department of Life Sciences and Bioengineering, Laboratory of Molecular and Cellular Biology, Faculty of Engineering, Academic Assembly, University of Toyama, 3190 Gofuku, Toyama 930-8555, Toyama, Japan; toza@eng.u-toyama.ac.jp; 7 Center for Advanced Antibody Drug Development, University of Toyama, 3190 Gofuku, Toyama 930-8555, Toyama, Japan

**Keywords:** vaccine, red sea bream iridovirus, high hydrostatic pressure, major capsid protein

## Abstract

Red sea bream (*Pagrus major*) aquaculture represents one of the most economically important marine aquaculture industries in Japan and East Asia. However, viral diseases, particularly those caused by red sea bream iridovirus (RSIV), pose a serious threat to aquaculture production in this region. In this study, we applied high-hydrostatic-pressure (HHP) refolding technology to develop a recombinant vaccine targeting the RSIV major capsid protein (MCP). The recombinant MCP (RSIV-rMCP) expressed in *Escherichia coli* was insoluble; however, HHP treatment under alkaline (pH 10) conditions in the presence of arginine successfully solubilised the protein while preserving its structural integrity. The solubilised protein (HHP–RSIV-rMCP) induced strong RSIV-specific IgM responses and enhanced disease resistance in red sea bream. In contrast, sera from fish immunised with a commercial formalin-inactivated vaccine exhibited minimal reactivity to HHP–RSIV-rMCP but reacted significantly to formalin-treated HHP–RSIV-rMCP. These results indicate that the HHP–RSIV-rMCP vaccine induces conformation-specific IgM antibodies and that structural preservation is crucial for maintaining antigenicity. Collectively, our findings demonstrate that HHP refolding technology is an effective strategy for preparing structurally preserved antigens.

## 1. Introduction

Aquaculture has become an essential component of the global fisheries industry, providing a sustainable source of high-quality protein for the growing human population [1]. In particular, aquaculture in Asia, including Japan, now accounts for a major share of total fishery production and continues to expand steadily [2]. However, intensive aquaculture practices create conditions that are highly susceptible to the spread of infectious diseases, which can cause substantial economic losses and compromise the sustainability of production [3,4]. Preventive vaccination is widely recognised as the most effective strategy for controlling infectious diseases in fish [5,6]. Therefore, the development of efficient and affordable fish vaccines is of growing importance for maintaining healthy aquaculture production. Among these infectious agents, iridoviruses (family *Iridoviridae*) are large double-stranded DNA viruses that cause severe systemic infections in a variety of marine and freshwater fish species, including red sea bream, and have led to major economic losses in aquaculture throughout East Asia [7,8].

Most commercial fish vaccines currently in use are formalin-inactivated whole-virus vaccines [9]. Although these vaccines are cost-effective and suitable for mass production, they often exhibit reduced antigenicity due to structural denaturation, leading to inconsistent protection [10]. In contrast, structure-preserved protein vaccines have demonstrated high immunogenicity and strong protective efficacy in human and veterinary medicine, highlighting the importance of maintaining native protein conformation for effective immune recognition [11,12]. Nevertheless, producing such structurally preserved antigens requires complex and costly purification processes, making them impractical for large-scale use in aquaculture, where cost constraints are critical [13]. Thus, there is a pressing need for a novel, low-cost method capable of preparing structurally preserved protein antigens suitable for fish vaccines.

High-hydrostatic-pressure (HHP) technology is a physicochemical method that enables protein refolding without chemical modification, thereby allowing recovery of the native conformation from denatured or aggregated states [14,15]. In recent years, HHP refolding has been experimentally applied to recombinant proteins and vaccine antigens for humans and animals, demonstrating its ability to restore structural and functional integrity [16,17]. Importantly, HHP-treated antigens have been shown to maintain conformational epitopes capable of eliciting conformation-recognising immune responses [6,15]. However, the application of HHP technology in fish vaccine antigen preparation has not yet been reported, despite its potential to preserve structural antigenicity and enhance immunogenicity. In this study, we attempted to utilize HHP technology for the preparation of fish vaccines.

## 2. Results

### 2.1. Preparation of RSIV-rMCP via High-Hydrostatic-Pressure Refolding

**Figure 1:**
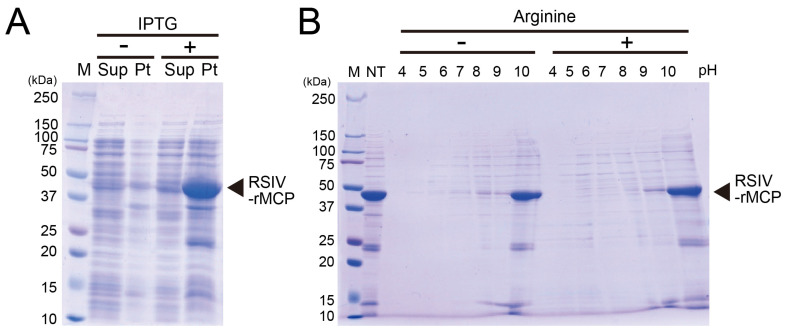
Preparation of high-hydrostatic-pressure-treated RSIV recombinant major capsid protein (HHP–RSIV-rMCP). (**A**) SDS–PAGE analysis of RSIV-rMCP expressed in *E. coli* after IPTG induction. M—molecular weight markers; Sup—supernatant fraction after cell sonication; Pt—pellet fraction after cell sonication. (**B**) SDS–PAGE analysis of the supernatant fraction of RSIV-rMCP after HHP treatment of the insoluble fraction. NT—non-treated insoluble fraction. Numbers above each lane indicate the pH value of the refolding buffer.

The recombinant major capsid protein of red sea bream iridovirus (RSIV-rMCP) was expressed in *Escherichia coli*. Upon induction with IPTG, RSIV-rMCP was produced efficiently; however, most of the expressed protein was localised in the pellet fraction, forming inclusion bodies (Figure 1A). To utilise RSIV-rMCP as a vaccine antigen, HHP refolding treatment was applied. Although HHP treatment alone did not achieve sufficient solubilisation, optimising buffer conditions showed that an alkaline environment (pH 10) markedly improved solubilisation efficiency (Figure 1B). Furthermore, the addition of L-arginine (Arg) to the pH 10 buffer further enhanced protein solubility, as confirmed via densitometric analysis of CBB-stained HHP–RSIV-rMCP bands, which showed an approximately 1.3-fold increase in soluble protein yield. These findings indicate that RSIV-rMCP expressed as inclusion bodies in *E. coli* was successfully solubilised using HHP treatment under alkaline (pH 10) conditions in the presence of arginine, and that the resulting HHP–RSIV-rMCP was subsequently employed for vaccine efficacy evaluation in red sea bream.

### 2.2. Evaluation of HHP–RSIV-rMCP Vaccine Efficacy

**Figure 2:**
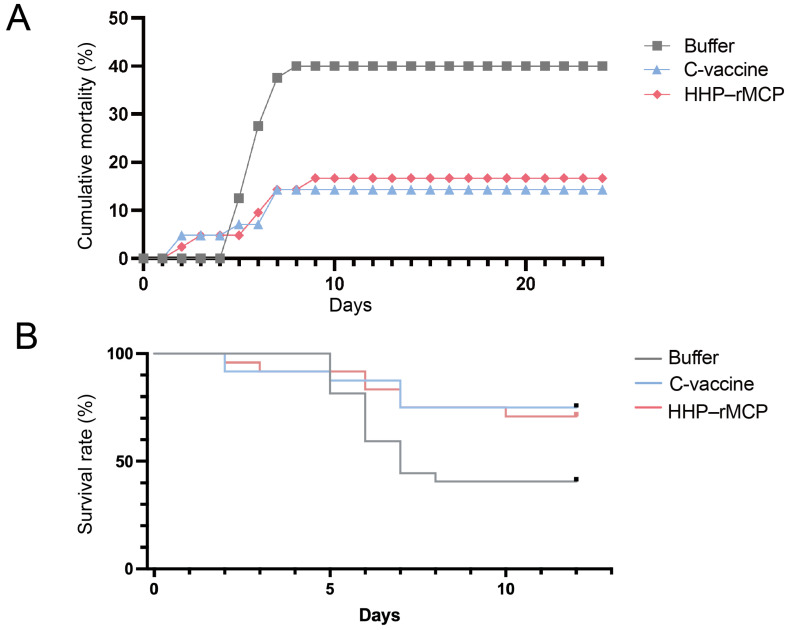
Evaluation of HHP–RSIV-rMCP vaccine efficacy. (**A**) Cumulative mortality after RSIV challenge in fish immunised with the buffer used for the HHP treatment (buffer, grey), commercial vaccine (C-vaccine, blue), or HHP-treated RSIV-rMCP (HHP–rMCP, red). (**B**) Kaplan–Meier survival analysis with log-rank testing derived from data shown in panel (**A**).

To evaluate the HHP–RSIV-rMCP vaccine efficacy, challenge and serological tests were conducted using juvenile red sea bream. Fish with an average body weight of 27.1 g were intraperitoneally injected with one of the following treatments: HHP–RSIV-rMCP, HHP treatment buffer (control), or a commercial vaccine. After the vaccination, the fish were reared for 20 days and subsequently challenged with RSIV. Mortality was monitored for 24 days post-infection in the HHP–RSIV-rMCP (*n* = 42), buffer (*n* = 40), and commercial vaccine (*n* = 42) groups. In the buffer control group, cumulative mortality began to increase sharply from day 5 post-infection, reaching 16 deaths and a final mortality rate of 40.0% (buffer in Figure 2A, grey squares). In the commercial vaccine group, fatalities were first observed on day 2 but remained low throughout the experimental period, resulting in six deaths and a final mortality rate of 14.3% (C-vaccine, blue triangles). The HHP–RSIV-rMCP-vaccinated group exhibited a comparable survival pattern, with seven deaths and a final mortality rate of 16.7% (HHP–rMCP, red diamonds). Kaplan–Meier survival analysis with log-rank testing further confirmed that vaccination with HHP–RSIV-rMCP significantly improved survival compared with the buffer control group throughout the post-challenge period (*p*-values for buffer vs. HHP-rMCP: 0.048; buffer vs. C-vaccine: 0.026; Figure 2B). These results indicate that administration of HHP–RSIV-rMCP confers disease resistance comparable with that provided by a commercially available vaccine in red sea bream.

### 2.3. Evaluation of HHP–RSIV-rMCP Immunogenic Activity

The vaccine evaluation experiments demonstrated that HHP–RSIV-rMCP conferred resistance to RSIV infection in red sea bream (Figure 2A). To determine whether this resistance resulted from an immune response specific to the HHP–RSIV-rMCP antigen, sera collected from each experimental group were analysed for the presence of IgM antibodies against HHP–RSIV-rMCP. Serum samples were obtained from red sea bream juveniles immunised with HHP–RSIV-rMCP, HHP treatment buffer (control), or the commercial vaccine (HHP–RSIV-rMCP group: *n* = 6; buffer group: *n* = 5; commercial vaccine group: *n* = 6). To minimise physiological stress caused by repeated sampling, sera were collected from a group of fish separate from those used in the challenge test.

**Figure 3:**
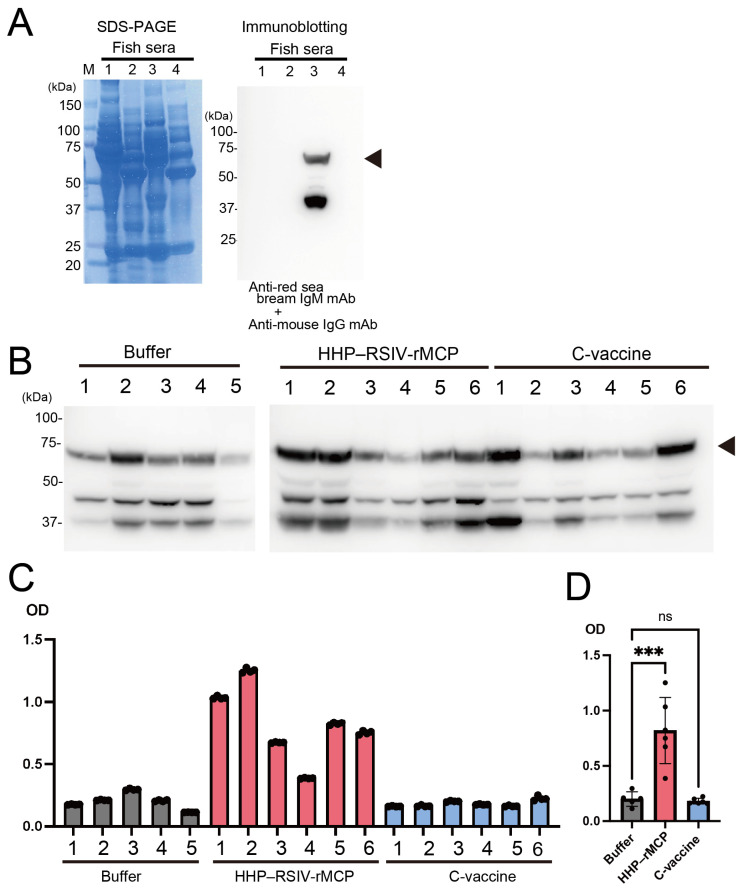
Evaluation of immune responses in red sea bream vaccinated with HHP–RSIV-rMCP. (**A**) Specificity analysis of the red sea bream IgM monoclonal antibody (mAb) derived from a hybridoma clone. Numbers indicate sera from the following fish species: 1—*Epinephelus septemfasciatus*; 2—*Scomber japonicus*; 3—*Pagrus major* (red sea bream); 4—*Trachurus japonicus*. The arrowhead indicates the IgM heavy chain. (**B**) Immunoblot analysis of red sea bream IgM using the anti-red sea bream IgM mAb described above. Numbers shown above each lane indicate individual fish from which serum samples were collected. The arrowhead indicates the IgM heavy chain. (**C**) ELISA results showing the binding activity of serum IgM to HHP–RSIV-rMCP in juvenile red sea bream immunised with the buffer used for HHP treatment (buffer, grey), HHP-treated RSIV-rMCP (HHP–RSIV-rMCP, red), or a commercial vaccine (C-vaccine, blue). Data are presented as mean ± SD (*n* = 4). (**D**) Mean absorbance values for each group shown in panel (**C**). Data are presented as mean ± SD. *** *p* < 0.0002.

To detect red sea bream IgM, a monoclonal antibody (mAb) that recognises the heavy chain (approximately 75 kDa) of red sea bream IgM (arrowhead in Figure 3A) was generated after the antibody gene was cloned from hybridoma cells. Immunoblot analysis using this mAb confirmed the presence of IgM in all serum samples, although variations in signal intensity were observed between individuals (Figure 3B). Subsequently, ELISA was conducted using the anti-red sea bream IgM mAb and HRP-conjugated anti-mouse IgG antibodies to assess the induction of HHP–RSIV-rMCP-specific IgM in each group. The ELISA results showed that sera from the buffer control group exhibited low absorbance values, whereas those from the HHP–RSIV-rMCP group demonstrated significantly higher values (Figure 3C). A positive correlation was observed between the ELISA values and total IgM levels detected via immunoblotting (Figure 3B,C). Interestingly, although the HHP–RSIV-rMCP and commercial vaccine groups exhibited similar protective effects in the challenge test (Figure 2A,B), sera from the commercial vaccine group showed significantly lower HHP–RSIV-rMCP-specific IgM reactivity (Figure 3D). These findings indicate that HHP–RSIV-rMCP effectively induces an IgM-mediated immune response in red sea bream, supporting its potential as a structurally preserved recombinant vaccine antigen.

### 2.4. Effect of Formalin Treatment on HHP–RSIV-rMCP Immunogenicity

**Figure 4:**
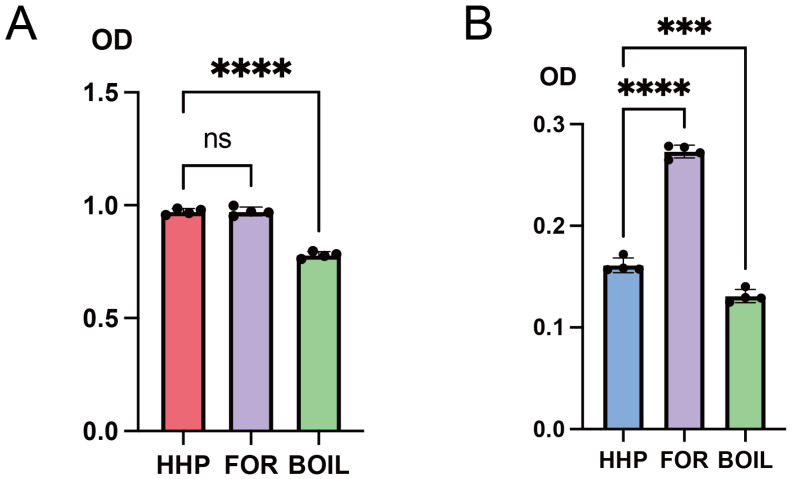
Evaluation of formalin-treated HHP–RSIV-rMCP reactivity using sera from disease-resistant red sea bream. (**A**) ELISA was used to assess the binding activity of IgM from red sea bream immunised with HHP–RSIV-rMCP to untreated HHP–RSIV-rMCP (HHP, red), formalin-treated (FOR, purple), or heat-treated (BOIL, green) antigens coated on the plate. (**B**) ELISA was used on sera from red sea bream immunised with a commercial vaccine, with plate-coated antigens of untreated HHP–RSIV-rMCP (HHP, blue), formalin-treated (FOR, purple), and heat-treated (BOIL, green) antigens. Data are presented as mean ± SD. *** and **** indicate *p* < 0.0002 and *p* < 0.0001, respectively. ns indicates no statistically significant difference (*p* > 0.05).

When evaluating the immunogenic activity of HHP–RSIV-rMCP, sera from the commercial vaccine group exhibited limited reactivity with HHP–RSIV-rMCP. This observation suggests that the immunogenic properties of the inactivated RSIV contained in the commercial vaccine differ from those of HHP–RSIV-rMCP prepared in this study. The principal distinction between these two antigens is that the commercial vaccine includes formalin-treated viral components. To examine the effect of formalin treatment, ELISA antigens were prepared by treating HHP–RSIV-rMCP with either formalin or heat. The reactivity of sera from the HHP–RSIV-rMCP group and the commercial vaccine group was then compared. When sera from the HHP–RSIV-rMCP group were tested, IgM reactivity toward formalin-treated and untreated HHP–RSIV-rMCP was similar; however, reactivity was significantly reduced against heat-treated HHP–RSIV-rMCP (Figure 4A). These findings suggest that heat denaturation disrupts conformational epitopes of HHP–RSIV-rMCP, resulting in a loss of recognition by IgM antibodies induced through the native antigen.

In contrast, when sera from the commercial vaccine group were analysed, IgM reactivity toward formalin-treated HHP–RSIV-rMCP was significantly higher than that toward the untreated form (Figure 4B). This result indicates that formalin treatment of HHP–RSIV-rMCP reproduces antigenic characteristics similar to those present in the formalin-inactivated RSIV contained in the commercial vaccine. Taken together, these findings demonstrate that the immunogenicity of RSIV-rMCP is strongly influenced by the antigen preparation method. Furthermore, the HHP-refolded RSIV-rMCP obtained in this study retains effective antigenic properties against RSIV, supporting its potential as a structurally preserved recombinant vaccine antigen.

## 3. Discussion

In this study, we examined high-hydrostatic-pressure (HHP) refolding as an antigen-processing strategy for generating a recombinant major capsid protein (MCP) of red sea bream iridovirus with preserved antigenic properties. Rather than evaluating vaccine efficacy, the present work focuses on how different antigen-processing methods influence antigenicity and immune recognition in teleost fish.

Immunological analyses demonstrated that HHP-refolded RSIV-rMCP induced IgM antibodies that preferentially recognised structurally preserved antigens and showed reduced reactivity toward heat-denatured MCP (Figure 4A). This pattern indicates that HHP refolding retains antigenic features sensitive to protein conformation. In contrast, sera from fish immunised with a formalin-inactivated commercial vaccine preferentially recognised formalin-treated antigens (Figure 4B), suggesting that chemical inactivation generates a distinct antigenic state of the same viral protein. These findings highlight qualitative differences in antibody recognition that arise from antigen-processing methods rather than differences in antigen sequence.

Although survival outcomes following RSIV challenge were comparable between groups, the infection model employed in this study was not designed to rigorously evaluate protective efficacy or to demonstrate superiority or non-inferiority. Accordingly, survival data are interpreted as supportive observations and not as definitive evidence of vaccine performance. The primary contribution of this study lies in the immunological characterisation of antigenicity rather than efficacy assessment.

It should also be noted that direct structural or biophysical analyses of HHP-refolded MCP were not performed. Consequently, conclusions regarding structural preservation are based on immunological evidence of conformation-dependent IgM recognition. Future studies incorporating direct structural characterisation and optimised infection models will be required to establish a more detailed molecular framework linking antigen structure, processing method, and immune outcome.

Although direct structural or biophysical analyses, such as circular dichroism spectroscopy or electron microscopy, were not performed in this study, the preservation of structure-dependent epitopes in HHP–RSIV-rMCP was indirectly supported through immunological evidence. Specifically, IgM induced via HHP–RSIV-rMCP recognised untreated and formalin-treated antigens but showed markedly reduced reactivity toward heat-denatured MCP (Figure 4A), indicating sensitivity to conformational disruption. These findings suggest that HHP treatment preserves higher-order structural features relevant to immune recognition. Nevertheless, direct structural characterisation of HHP-refolded MCP will be an important focus of future studies to further substantiate these observations.

Although vaccination with HHP–RSIV-rMCP and the commercial formalin-inactivated vaccine resulted in comparable survival rates following RSIV challenge, the antibody recognition profiles induced via these two vaccines were clearly distinct. The HHP–RSIV-rMCP vaccine preferentially elicited IgM antibodies that recognised the untreated, structurally preserved recombinant MCP (Figure 4A), whereas sera from fish immunised with the commercial vaccine exhibited stronger reactivity toward formalin-treated antigens (Figure 4B). These findings suggest that comparable levels of protective efficacy can be achieved through immunologically distinct pathways, depending on the structural characteristics of the antigen used for vaccination. In this context, protection may not solely depend on the quantity of antigen-specific antibodies but also on qualitative differences in epitope recognition and antigen–antibody interactions.

Notably, vaccination with HHP–RSIV-rMCP and the commercial formalin-inactivated vaccine resulted in comparable survival rates despite clear differences in IgM recognition profiles. This observation suggests that similar protective outcomes can arise from immunologically distinct response patterns. While the present study focused on the induction of antigen-specific IgM, protection against RSIV infection in teleost fish is likely mediated through multiple immune components. In addition to humoral IgM responses, innate antiviral mechanisms, including interferon-related pathways, phagocytic activity, and complement activation, as well as cellular immune responses, have been reported to contribute to antiviral defence in fish [18]. Furthermore, antibodies recognising structurally altered or conserved viral components may exert protective effects through mechanisms other than direct neutralisation, such as opsonisation or modulation of viral dissemination [19,20]. Therefore, the comparable protection observed in this study likely reflects the combined contribution of multiple immune pathways, with antigen structure influencing the qualitative profile of the humoral response rather than solely determining overall protective efficacy.

Dose–response experiments were not performed in the present study; however, such studies are planned for future investigations to further optimise the vaccination protocol.

## 4. Materials and Methods

### 4.1. Recombinant RSIV-MCP Preparation

The DNA encoding the major capsid protein (MCP, Q80M45_RSIV) of red sea bream iridovirus (RSIV) was synthesised via GenScript using *Arabidopsis* codon usage frequency. After the addition of restriction enzyme sites via PCR, the fragment was ligated into the pET-30a vector using a Ligation High ver. 2 enzyme (Toyobo, Osaka, Japan). *E. coli* Rosetta 2 (DE3) cells transformed with the expression plasmid were pre-cultured overnight at 37 °C in LB medium containing 50 µg/mL kanamycin. On the following day, after measuring the optical density at 600 nm (OD_600_) of the pre-culture, a volume sufficient to achieve an OD_600_ of 0.04 in LB medium containing kanamycin was inoculated. The culture was shaken at 37 °C until the OD_600_ reached 0.3, after which the incubator temperature was reduced to 10 °C. Shaking continued while the temperature was gradually lowered until the OD_600_ reached 0.6, at which point expression was induced. Expression was induced with 0.25 mM isopropyl β-D-thiogalactopyranoside (IPTG), followed by shaking at 20 °C for 20 h. Cells were harvested via centrifugation at 4 °C, 8000 rpm for 5 min. The resulting cell pellet was resuspended in A buffer (50 mM Tris-HCl, pH 8.0, 50 mM NaCl, 0.5 mM EDTA, 5% glycerol, 1 mM TCEP), and the *E. coli* cells were lysed. After disruption, inclusion bodies (IBs) were recovered via centrifugation at 4 °C and 15,000× *g* for 30 min. Recombinant protein expression was confirmed using SDS-PAGE. Each IB preparation was washed once with 10 mL of B buffer (A buffer + 0.125 M NDSB-201) per 1 g of IB, followed by two washes with an equal volume of A buffer. After each wash, the IB suspension was centrifuged at 8000× *g* for 15 min at 10 °C, and the supernatant was discarded. The purified IB was resuspended in an equal volume of Milli-Q water and stored at −80 °C until use. The recombinant MCP was prepared from inclusion bodies and subjected to multiple washing steps prior to solubilisation and refolding, which was expected to reduce endotoxin contamination. However, endotoxin levels were not directly measured in this study.

### 4.2. HHP Refolding

One millilitre of IB suspension was diluted with 9 mL of refolding buffer (50 mM CAPS–NaOH, pH 10.0, 0.5 M L-arginine), mixed thoroughly, and injected into a polypropylene Bell-top Quick-Seal centrifuge tube (Beckman Coulter, Brea, CA, USA), which was then sealed. The following buffers were used as refolding buffers for various pH adjustments: 50 mM acetate, pH 4.0–5.0; 50 mM MES, pH 6.0; 50 mM HEPES, pH 7.0; 50 mM Tris, pH 8.0; 50 mM CHES, pH 9.0; 50 mM CAPS, pH 10.0. Refolding was performed using a PreEMT-E150 pressure chamber (BaroFold Inc., Boulder, CO, USA). The pressure was increased to 200 MPa and maintained for 16 h at room temperature. After depressurisation at a rate of 25 MPa per 5 min, the sample was transferred to a microtube and centrifuged at 20,000× *g* for 30 min at 4 °C. Following buffer replacement (PBS + 0.5 M arginine) using PD-10 columns (Cytiva, Marlborough, MA, USA), the resulting HHP–RSIV-rMCP was stored at −80 °C until used as the immunogen for fish. Refolding efficiency was assessed using SDS–PAGE and visualised by staining with Coomassie Brilliant Blue (CBB) R-250.

### 4.3. Fish

Juvenile red sea bream (*Pagrus major*) with an average body weight of 27.1 g were obtained from the Marua Suisan Co., Ltd. (Kamijima Town, Ehime, Japan). Before the experiments, the fish were acclimated for seven days in tanks containing running seawater and were fed commercial pellets as appropriate. To confirm the absence of active RSIV infection in the experimental fish population, ten fish were randomly sampled from the same stock prior to the experiments. DNA was extracted from spleen tissues and analysed via real-time PCR according to the method described in a previous study [21]. All tested samples were confirmed to be RSIV-negative.

### 4.4. Vaccination, RSIV Infection, and Serum Preparation

HHP–RSIV-rMCP was resuspended in PBS containing 0.5 M L-arginine (PBS + 0.5 M Arg) at a concentration of 1 mg/mL before use. Red sea bream (*n* = 20 per group) were anaesthetised with phenoxyethanol and intraperitoneally injected with 0.2 mg of HHP–RSIV-rMCP per fish. Phenoxyethanol used for fish anaesthesia was purchased from FUJIFILM Wako Pure Chemical Corporation (Osaka, Japan) and was administered at a final concentration of 300 ppm according to the method described in a previous study [22]. Negative control fish were injected with PBS + 0.5 M Arg alone, while positive control fish were injected with 100 µL of a commercial vaccine (Biosiense Co., Ltd., Tokushima, Japan). The commercial vaccine used in this study was Marine Jenner Irido. The infectious titre prior to inactivation was ≥10^9.1^ TCID_50_ per 100 mL, corresponding to ≥10^6.1^ TCID_50_ per fish dose. The vaccine dose used in this study (0.2 mg per fish) was selected based on a previous report in which a fish vaccine was administered at a dose of 200 µg per fish [23]. Fish were maintained in 180 L tanks at a constant water temperature of 22 °C. RSIV was prepared from spleen tissues of diseased red sea bream, yellowtail (*Seriola quinqueradiata*), and striped jack (*Pseudocaranx dentex*) collected in Ehime Prefecture in 2021, all of which were confirmed to be RSIV-positive according to real-time PCR. A preliminary infection trial was conducted, and immunised fish were challenged with a viral inoculum that produced approximately 50% mortality at 20 days post-vaccination. Mortality was recorded twice daily for 20 days. Serum samples were collected from each group before viral challenge and stored at −80 °C until use. In this study, separate fish populations were used for serological analyses and viral challenge experiments. This experimental design was chosen to minimise handling-related stress, as fish subjected to blood collection were considered unsuitable for subsequent challenge experiments.

### 4.5. Monoclonalisation of Anti-Red Sea Bream IgM Antibody

The amplification of antibody cDNA from hybridoma cells and the monoclonalisation of anti-red sea bream IgM antibody were carried out using the same methods as previously described [16].

### 4.6. Immunoblotting

Immunoblotting and CBB staining were performed according to standard methods described previously [24]. Protein samples were separated via SDS-PAGE. For CBB staining, the SDS-PAGE gel was stained with CBB and subsequently destained through several washes with water. In particular, the SDS-PAGE gel was transferred onto polyvinylidene difluoride (PVDF) membranes (#IPVH00010, Millipore, Bedford, MA, USA) for immunoblot analysis. The membranes were blocked using 5% skim milk (#4273437, Megmilk Snow Brand, Tokyo, Japan) in TBST (20 mM Tris-HCl [pH 7.5], 150 mM NaCl, 0.05% Tween20) at 27 °C for 1 h, and then treated with the appropriate antibodies. Immobilon (#WBKLS0500, Millipore) was used as a substrate for HRP, and the luminescence signal was detected using an ImageQuant LAS 4000 mini (version 1.1, GE Healthcare, Camarillo, CA, USA).

### 4.7. Enzyme-Linked Immunosorbent Assay (ELISA)

The ELISA was conducted using MaxiSorp 96-well immunoplates (NUNC, Thermo Fisher Scientific, Waltham, MA, USA). The plates were washed three times with Tris-buffered saline containing 0.5% Tween 20 (TBST) at room temperature. A 100 µL aliquot of HHP–RSIV-rMCP (2 µg/mL in PBS) was added to each well and incubated at 4 °C overnight for coating. After blocking with 5% skimmed milk in TBST, red sea bream (*Pagrus major*) serum samples diluted 1:500 in PBS were incubated for 2 h at room temperature. After washing, an anti-IgM mouse monoclonal antibody diluted 1:500 in PBS was added and incubated overnight at 4 °C. Following additional washes, horseradish peroxidase (HRP)-conjugated anti-mouse IgG antibody (#7076, Cell Signaling Technology, Danvers, MA, USA) diluted 1:1000 in PBS was added and incubated for 2 h at room temperature. The presence of IgM was detected via incubation with the HRP substrate (1-Step Ultra TMB-ELISA, Thermo Fisher Scientific) for 5 min before adding 1 M HCl as the stop solution. Absorbance was measured at 450 nm using a SpectraMax M3 plate reader (Molecular Devices, San Jose, CA, USA). For antigen preparation using formalin treatment, HHP–RSIV-rMCP was treated with 0.2% formalin in PBS for four days at 4 °C and used for the assay under the same conditions. Heat treatment was carried out at 98 °C for 5 min.

### 4.8. Statistical Analysis

Data were expressed as mean ± standard deviation (SD). Multiple pairwise comparisons were performed using one-way analysis of variance followed by Dunnett’s test, implemented in GraphPad Prism version 10.6.1. ImageJ software (version 1.53t, National Institutions of Health, Bethesda, MD, USA) was used to quantify RSIV-rMCP band intensity.

## 5. Conclusions

This study shows that high-hydrostatic-pressure (HHP) refolding enables the preparation of a soluble recombinant major capsid protein of red sea bream iridovirus while preserving conformation-dependent antigenicity. HHP-refolded RSIV-rMCP induced IgM antibodies that preferentially recognised structurally preserved antigens, whereas sera from fish immunised with a formalin-inactivated commercial vaccine preferentially recognised formalin-treated antigens, indicating that different antigen-processing methods generate distinct antigenic states. Although direct structural analyses were not performed, these findings establish HHP refolding as an antigen-processing strategy that preserves antigenic properties distinct from those produced by chemical inactivation.

## Data Availability

The data used to support the findings of this study are available from the corresponding authors upon request.

## Abbreviations

RSIV—red sea bream iridovirus; HHP—high-hydrostatic-pressure; rMCP—recombinant major capsid protein; ELISA—enzyme-linked immunosorbent assay; mAb—monoclonal antibody.
